# Mouse Embryonic Stem Cell-Derived Ureteric Bud Progenitors Induce Nephrogenesis

**DOI:** 10.3390/cells9020329

**Published:** 2020-01-31

**Authors:** Zenglai Tan, Aleksandra Rak-Raszewska, Ilya Skovorodkin, Seppo J. Vainio

**Affiliations:** 1 Center for Cell Matrix Research, Faculty of Biochemistry and Molecular Medicine, Biocenter Oulu, Laboratory of Developmental Biology, Infotech Oulu, University of Oulu, Aapistie 5A, 90220 Oulu, Finland; Aleksandra.Rak-Raszewska@oulu.fi (A.R.-R.); ilya.skovorodkin@oulu.fi (I.S.); 2 Borealis Biobank of Northern Finland, Oulu Central Hospital, 90220 Oulu, Finland

**Keywords:** mouse embryonic stem cell, differentiation protocol, ureteric bud progenitor cells, 3D kidney organoids

## Abstract

Generation of kidney organoids from pluripotent stem cells (PSCs) is regarded as a potentially powerful way to study kidney development, disease, and regeneration. Direct differentiation of PSCs towards renal lineages is well studied; however, most of the studies relate to generation of nephron progenitor population from PSCs. Until now, differentiation of PSCs into ureteric bud (UB) progenitor cells has had limited success. Here, we describe a simple, efficient, and reproducible protocol to direct differentiation of mouse embryonic stem cells (mESCs) into UB progenitor cells. The mESC-derived UB cells were able to induce nephrogenesis when co-cultured with primary metanephric mesenchyme (pMM). In generated kidney organoids, the embryonic pMM developed nephron structures, and the mESC-derived UB cells formed numerous collecting ducts connected with the nephron tubules. Altogether, our study established an uncomplicated and reproducible platform to generate ureteric bud progenitors from mouse embryonic stem cells.

## 1. Introduction

Pluripotent stem cells (PSCs) possess great potential of differentiating into multiple cell types that are widely used for studies in developmental biology and regenerative medicine [1]. Kidney organoids derived from PSCs have been shown to be able to mimic the in vivo kidney structure development and function in vitro [2,3,4]. Renal organoids in a four-dimensional (4D) (3D plus time) culture system self-organize into highly complex tissue-specific morphology that is sufficient to model tissue development, disease, and injury [5,6,7,8]. A combination of genome editing and stem cell technologies allows for generation of personalized kidney organoids, which provide powerful tools for kidney disease treatment, drug toxicity screening, and tissue regeneration [9,10].

Recently, multiple protocols enabling generation of renal lineages from mouse and human PSCs have been published. We and several other groups reported induction of nephron progenitors, which have the potential to develop into epithelial nephron-like structures [2,3,4,8,11,12,13,14,15,16,17,18]. Other groups have shown the derivation of ureteric bud (UB) progenitors [19]. However, they did not show nephron progenitor induction and also lacked the connection between collecting ducts and nephrons [2,19]. A newly published study has shown generation of UB structures from PSCs, which possessed UB-like branching morphogenesis when aggregated with the primary metanephric mesenchyme (pMM) to form chimeric kidney organoids [8]. However, the protocol is technically complex, which limits its application. Analysis of these reports suggests that we still need further studies to develop simple, reproducible, and stable protocols for UB progenitor generation.

Here, we report a simple protocol to direct differentiation of mouse embryonic stem cells (mESCs) into UB progenitor cells. The newly generated UB progenitor cells have a potential to develop into ureteric bud structures and have a capacity to induce nephrogenesis when co-cultured with dissociated pMM. In reconstructed kidney organoids, the pMM developed into nephron structures and the UB progenitor cells formed collecting ducts, which also connected with the nephron tubules. The chimeric kidney organoids also display the presence of endothelial cells forming a vascular network. In conclusion, our study established an uncomplicated and reproducible method for generation of UB progenitors from PSCs that can be used for tubulogenesis induction.

## 2. Materials and Methods

Animal care and experimental procedures in this study were in accordance with Finnish national legislation on the use of laboratory animals, the European Convention for the protection of vertebrate animal used for experimental and other scientific purposes (ETS 123), and the EU Directive 86/609/EEC. Animal experimentation was also authorized by the Finnish National Animal Experiment Board (ELLA) as being compliant with the EU guidelines for animal research and welfare.

### 2.1. Mouse ES Cell Line Generation and Maintenance

mESC line Sv129S6 was obtained from the Biocenter Oulu Transgenic core facility. The Sv129S6 mESC line was derived from Taconic’s W4/129S6 inbred mouse strain and has been tested to have a normal karyotype [19]. The mESC and mESC-GFP (Green Fluorescent Protein, pcDNA3.1 transfected) line was described previously [20]. It was maintained on mitotically inactivated mouse embryonic fibroblasts (MEFs) in mESC medium: Dulbecco’s Modified Eagle’s Medium (DMEM; Life Technologies, Waltham, MA, USA) supplemented with 10% fetal bovine serum (FBS, Gibco, Waltham, MA, USA ), 1% (*v*/*v*) nonessential amino acids (Life Technologies), 100 mM 2-mercaptoethanol (Nacalai Tesque, Kyoto, Japan), and 1000 U/mL leukemia inhibitory factor (LIF, Millipore, Espoo, Finland).

### 2.2. Directed Differentiation of mESCs to UB Progenitors

Mouse ESCs were cultured as described above in MEF-coated 6-well plates in mESCs medium up to 70%–90% confluency. The cells were passaged on 1% geltrex-coated 24-well plates at 30,000 cells/cm^2^ in 500 μL 50% ReproFF2 (ReproCELL, Glasgow, UK)—50% CM (conditioned MEF medium) supplied with 10 ng/mL fibroblast growth factor (FGF) 2 (PeproTech Nordic, Stockholm, Sweden) and 10 ng/mL Activin A (Cell Guidance Systems, Cambridge, UK). After 2 days, the cells reached 60–80% of confluency, and the medium was changed to differentiation medium: Advanced RPMI (Thermo Fisher Scientific, Waltham, MA, USA) supplemented with 8 μM CHIR99021 (Reagents Direct, Encinitas, CA, USA) and 4–8 ng/mL Noggin (R&D systems, Minneapolis, MN, USA) for 2 days, followed by treatment of the cells with Activin A (10 ng/mL) for 2 days and then 3 days with FGF9 (40 ng/mL) (PeproTech Nordic, Stockholm, Sweeden) in Advanced RPMI medium (ThermoFisher Scientific).

### 2.3. Gene Expression Analysis

A RNeasy kit (Qiagen, Germantown, MD, USA) was used according to the manufacturer’s recommendations to extract total RNA. cDNA synthesis (First Strand cDNA Synthesis Kit, ThermoFisher Scientific, Waltham, MA, USA) was performed using standard protocols. qPCR was run using a CFX96 BioRad thermocycler. Brilliant III SYBR^®^ Green QPCR Master Mix (Agilent Technologies, Santa Clara, CA, USA) was used according to the manufacturer’s instructions. GAPDH probe served as a control to normalize the data. The data was analyzed using the 2^−∆∆CT^ method. Gene expression measurements were performed in triplicates on three independent experiments. The primer sequences are given in Supplementary Table S1.

### 2.4. Chimera 3D Kidney Organoids Formation

At day 9 of mESCs differentiation, which represents the UB progenitor cells stage, cells were dissociated into single cell suspension using TrypLE select (Life Technologies). After three washes in phosphate buffer saline (PBS), cells were reconstituted in organ culture medium (DMEM supplemented with 10% fetal bovine serum, 1% penicillin–streptomycin). For generation of kidney organoids, we mixed dissected and dissociated pMM cells (from CD-1 pregnant females, at E11.5), as described before [21], with differentiated UB progenitors at a ratio of 3:1, respectively. Briefly, kidney rudiments were dissected out of E11.5 embryos and treated with trypsin/pancreatin solution for 30–40 s to separate the UB from the pMM [22]. The MM cells were dissociated using 2.0 mg/mL Collagenase IV solution (Wortington, Lakewood, NJ, USA) in 0.1% bovine serum albumin (BSA) in 1×PBS. 10 min incubation was interrupted by pipetting and continued until cells were separated. The reaction was stopped by washing three times in complete organ culture medium [22]. The pMM- and mESC-derived UB cells were mixed in a 3:1 ratio and centrifuged for 4 min at 1380× *g* to form a pellet (5 × 10^4^ cells) in Lo-Binding Eppendorf tubes. Following centrifugation, we carefully transferred the differentiated UB and pMM pellets to filter into Trowel culture to aggregate as an organoid. The organ culture medium was changed every 3–4 days.

For generation of whole kidney organoids, we dissected mouse kidney rudiments at E11.5 from CD-1 pregnant females. Kidney rudiments were dissociated into single cell suspension as described previously [19]. After dissociation, the embryonic kidney cells (7 × 10^4^) were mixed with undifferentiated mESC or differentiated mESCs-derived UB progenitors (1 × 10^4^) to make the pellet. We then continued the procedure as described above.

### 2.5. Whole-Mount Immunohistochemistry

Kidney organoids were washed 2 times with PBS and fixed with 100% cold methanol (–20 °C) for 30 min at room temperature (RT) or with 4% paraformaldehyde in PBS (organoid with GFP or dye) for 30 min at RT in the dark. After fixation, the organoids were washed at least three times in PBS and blocked in 0.1% Triton-X100 (Sigma, Lyon, France), 1% BSA, and 10% goat serum/0.02M glycine-PBS for 1–3 h at room temperature. Incubation of the organoids with primary antibodies was performed in a blocking buffer overnight at 4 °C. The samples were washed 6 times with PBS and incubated with secondary antibodies Alexa Fluor 405, 488, 568, 546, or 647 (1:1000, Life technologies) and fluorescein anti-LTL (Lotus Tetragonolobus Lectin, 1:350, #FL-1321, Vector Laboratories, Burlingame, CA, USA) overnight at 4 °C and counter-stained with Hoechst (Thermo Fisher Scientific). The primary antibodies used in stainings were: Wt1 (1:100, #05-753, Millipore), Pax2 (1:200, #PRB-276P, Covance, Cambridge, MA, USA), Troma1 (1:200, DSHB, Iowa City, IA, USA), Gata3 (1:20, #AF2605-SP, R&D Systems), E-cad (1:300, #610181, BD Biosciences, Franklin Lakes, NJ, USA), Synaptopodin (SYNPO) (1:4, #ABIN112223, antibody on line.com, Aachen, Germany), Umod (1:25, #LS-C150268, LSBio, Seattle, WA, USA), CD31 (1:100, #550274 BD Biosciences), Laminin (1:200, #L9393, Sigma), and Cleaved Caspase-3 (1:200, #9661s, Cell Signaling Technology, Leiden, Netherlands). Stained organoids were mounted with Shandon™ Immu-Mount™ (Thermo Scientific™). A Zeiss LSM780 microscope and Zeiss Axiolab (Zeiss, Oberkochen, Germany) were used for image capture and analysis.

### 2.6. Nephrotoxicity Assay

3D kidney organoids were cultured in organ culture medium supplemented with gentamicin at 5 mg/mL (#G1264, Sigma) for 48 h, or with cisplatin at 5, 20, or 50 µM (#P4394 Sigma) for 24 h after day 8 of organ culture. Organoids were then fixed with 100% cold methanol for 30 min for whole-mount immunohistochemistry. The Notch inhibitor, *N*-*S*-phenyl-glycine-*t*-butyl ester (DAPT, #D5942, Sigma), was used (10µM) to investigate toxicity towards proximal tubule development.

### 2.7. Statistical Evaluation

All data are presented as mean ± standard deviation (SD) and represent a minimum of three independent experiments. Student’s two-tailed t-test was used for statistical evaluation. *p*-value < 0.05 was considered significant.

## 3. Results

### 3.1. Direct Differentiation of mESCs into UB Progenitor Cells

**Figure 1:**
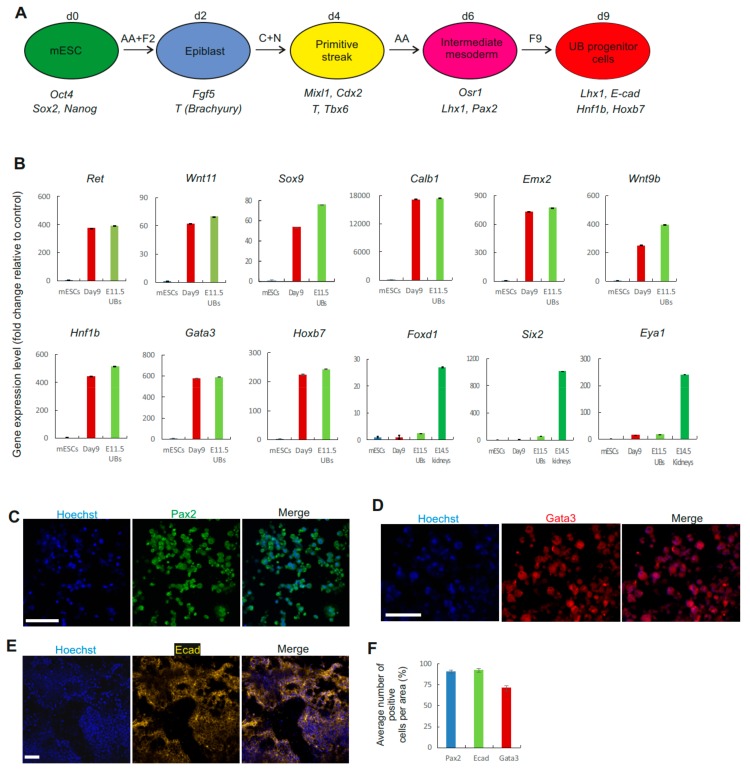
Differentiation of mouse embryonic stem cells (mESCs) to ureteric bud (UB) progenitor cells. (**A**) Schematic of the differentiation protocol of mESCs into UB progenitor cells. AA: Activin A; F2: FGF2; C: CHIR99021; N: Noggin; F9: FGF9. (**B**) Graphs of qPCR results showing the gene expression (fold change) of ureteric bud markers relative to mESC. No expression of stroma (Foxd1) and nephron progenitor cell markers (Six2 and Eya1) was observed at day 9 of differentiation. (*n* = 3). (**C**–**E**) Immunocytochemistry of Pax2, Ecad, and Gata3 in mESCs on day 9 of differentiation. Scale bars, 50 μm. (**F**) Quantification of the number of cells expressing Pax2, Ecad, and Gata3 at day 9 of differentiation. *n* = 3 samples per marker (3 randomly chosen areas in 3 independent experiments).

During development, both the nephron and ureteric bud progenitor cells are derived from the intermediate mesoderm (IM). To establish a protocol and direct differentiation of mESCs into UB lineage, we first differentiated mESCs into IM (Figure 1A). We treated the mESCs with FGF2 and activin A to differentiate mESCs into epiblast in monolayer culture (Supplementary Figure S1A). The differentiated cells showed expression of epiblast markers such as Fgf5 and T (Brachyury) (Supplementary Figure S1B). We then used glycogen synthase kinase-3β ( GSK-3β) inhibitor, CHIR99021 (CHIR), together with a low concentration of bone morphogenetic protein (BMP) signaling inhibitor, Noggin (suppresses number of cells differentiated to lateral plate mesoderm) [2], to activate differentiation of mouse epiblast cells into primitive streak (PS). The Noggin-treated cells expressed PS markers Cdx2, T, Tbx6, and Mixl1 (Supplementary Figure S1B). Activin A has been previously used for specification of the mesodermal cells towards intermediate mesoderm [19]. Therefore, we followed for two days with activin A treatment, which differentiated the cells to the IM stage and expressed Osr1, Lhx1, and Pax2 (Supplementary Figure S1B).

Previous studies have demonstrated that FGF9 is able to induce renal lineage differentiation from the IM population [2]. Therefore, we treated these cells with a moderate concentration of FGF9 for an additional three days, directing them to differentiate into UB progenitor cells with expression of UB markers. These cells expressed UB tip markers: Ret, Wnt11, and Sox9, as well as other markers of UB: Lhx1, Ecad, Hnf1b, Wnt7b, Wnt9b, Calb1, Emx2, Gata3, Hoxb7, and Tacstd2 (Figure 1B and Supplementary Figure S1C). In addition, expression of stromal cell marker Foxd1 nephron progenitor cell markers, Six2 and Eya1 (Figure 1B), or other epithelial segment markers, were observed at day nine of differentiation (Supplementary Figure S1D). Immunofluorescence staining further revealed that the use of a moderate concentration of FGF9 induced the cells to express Pax2, E-cadherin (Ecad), and Gata3 (Figure 1C–F), which may suggest that these differentiated cells represent putative UB progenitor cells.

### 3.2. Generation of Kidney Organoids by mESC-Derived UB Progenitor Cells and Dissociated Primary MM Population

**Figure 2:**
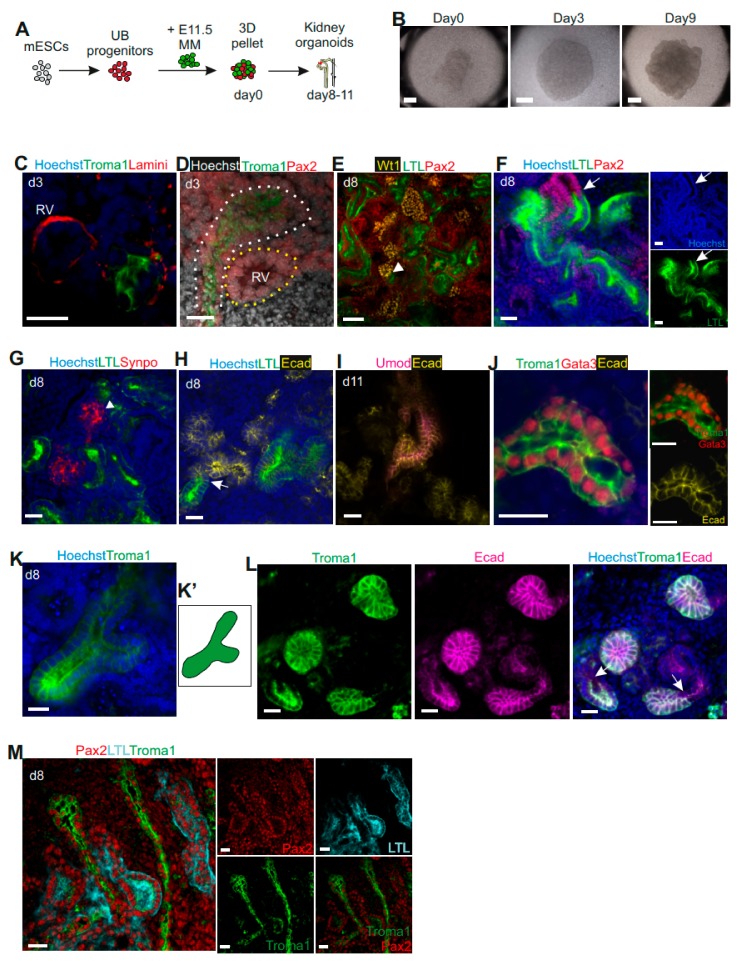
Generation of renal organoids by mESC-derived UB progenitors and primary metanephric mesenchyme (pMM) cells. (**A**) Schematic of generation of kidney organoids from mESC-derived UB progenitors with mouse E11.5-dissociated pMM. (**B**) Global bright field images of self-organizing kidney organoids in a Trowel organ culture system. Scale bars, 500 μm. (**C**) Immunofluorescence of kidney organoids at day 3 show formation of a renal vesicle next to the Troma1+ structure generated by mESC-derived UB progenitor cells. (**D**) Confocal image at day 3 showing Pax2+ renal vesicle (yellow dotted line) surrounded by Troma+Pax2+ ureteric epithelium (white dotted line). (**E**–**M**) Immunofluorescence of kidney organoids at day 8 or 11. (**E**) Glomeruli are marked with Wt1, proximal tubules are marked with LTL, and distal tubules are marked with Pax2+LTL–. The arrowhead shows the connection location of glomeruli with proximal tubule. (**F**) Immunostaining of distal tubule marked with Pax2 and proximal tubule marked with LTL, with all nuclei stained with DAPI. The arrow shows the proximal tubules (Pax2+LTL+) connected with distal tubules (Pax2+LTL–). (**G**) Glomeruli (Synpo+) adjacent to proximal tubules (LTL+) (the arrowhead marks the place of connection). (**H**) Immunostaining of proximal tubule with LTL and distal tubule with Ecad. Confocal image shows proximal tubules (LTL+) connected with distal tubules (Ecad+LTL–) (marked with an arrow). (**I**) Confocal image of Loops of Henle marked by Umod and Ecad on day 11. (**J**) Confocal images of Troma1+Gata3+Ecad+ collecting duct structure. (**K-K’**) A “T” shaped UB structure in the kidney organoid. (**L**) mESC-derived UB progenitor cells-generated collecting ducts (Troma1+Ecad+) connecting with nephron’s distal tubules (Troma1–Ecad+) (marked with an arrow). (**M**) Kidney organoids developed collecting duct trunk structures. Scale bars, (**B**) 500 μm, (**E**) 50 μm, (**C**–**D**, **F**–**M**) 20 μm.

We and other groups previously reported that dissociation of mouse pMM into single cells maintains the nephron progenitor stemness. The dissociated MM population develops into nephrons when induced by the inducer such as the embryonic UB or spinal cord cells [8,21,23,24,25,26,27]. To establish the potential and function of the mESC-derived UB progenitor cells, we aggregated these cells with mouse E11.5-dissociated pMM cells to generate a kidney organoid. The cell aggregates were cultured for up to 11 days in a traditional Trowell organ culture system, during which they spontaneously formed kidney organoids with complex structures (Figure 2A,B). On day three, we observed Troma1+ UB structures and the formation of renal vesicles adjacent to the UB (Figure 2C–D).

Whole-mount immunostaining of day eight chimeric organoids showed development of nephrons with positive staining of glomerular markers Wilms tumor 1 (Wt1+) and Synaptopodin (Synpo+), proximal tubule marker Lotus Tetragonolobus Lectin (LTL+), and distal tubules markers Pax2+LTL– and Ecad+ (Figure 2E–H, Supplementary Figure S2A). Moreover, we found numerous Synpo+ and Wt1+ glomeruli adjacent to LTL+ proximal tubules, which connected with Pax2+LTL–/Ecad+ distal tubules (Figure 2E–H marked with arrowheads and arrows, Supplementary Figure S2A), indicating a proper nephron structure with glomerulus/proximal tubule/distal tubule organization. On day 11, the organoids also displayed Henle’s loop with the expression of uromodulin (Umod+) and Ecad+ (Figure 2I).

We also tested whether the mESC-derived UB progenitor cells have in vivo UB capacity to form the collecting duct system. On day eight, we observed Troma1+Gata3+Ecad+Pax2+LTL– collecting duct structures in the kidney organoids by immunocytochemistry (Figure 2J–M, Supplementary Figure S2B). We also found some Troma1+ “T” shaped UB structures (Figure 2K, K’), indicating that the mESC-derived UB progenitors behave in a manner similar to UB cells in vivo. Importantly, we observed that the collecting ducts (Troma1+Ecad+) connected with the distal tubules (Ecad+Troma1–) of the nephron structure (Figure 2L, Supplementary Figure S2C), generating an interconnection between collecting ducts and nephrons, which is essential for urine drainage. The collecting ducts displayed the morphology of branches and long collecting duct trunks (Figure 2M). Altogether, the *in vitro* reconstructed organoids developed kidney structures that are similar to in vivo kidney, although it is unclear if the generated UB structures form a proper network.

We have also attempted to induce vascularization of developing glomeruli in these renal organoids. Previous studies demonstrated that rho-associated protein kinases (ROCK) are downstream effectors of vascular endothelial growth factor (VEGF), and negatively regulate the process of angiogenesis [28]. Therefore, we used the ROCK inhibitor to enhance angiogenesis in the renal organoids. However, this treatment did not increase the endothelial network area, and CD31+ cells could not be found in the developing glomerular tuft (Supplementary Figure S3).

### 3.3. Characterization of Kidney Organoids

To rule out the possibility that the differentiated cells (mESC-derived UB progenitors) could give rise to nephrons when induced by the embryonic UB, we aggregated day nine differentiated cells with E11.5-dissociated UB cells and grew them in organ culture. Since UB survival and development depend on the presence of metanephric mesenchyme, in the organ cultures, the purified UB failed to branch in the presence of UB-differentiated cells (Supplementary Figure S4A). Next, we wanted to verify that nephron structures in the organoids were generated via the MM population induced by the mESC-derived UB progenitors, and not by contamination of the MM with the primary UB cells. To assess this possibility, we cultured E11.5 MM tissue in isolation. The tissue already underwent apoptosis at the second day of culture and died at day three (Supplementary Figure S4B). This result also confirmed that without a suitable inducer, the MM cells will not undergo nephrogenesis and cannot survive for a long time under the organ culture conditions in vitro. These data suggest that we did differentiate mESC towards UB progenitors, and that they can further develop into collecting ducts when co-cultured with pMM.

**Figure 3:**
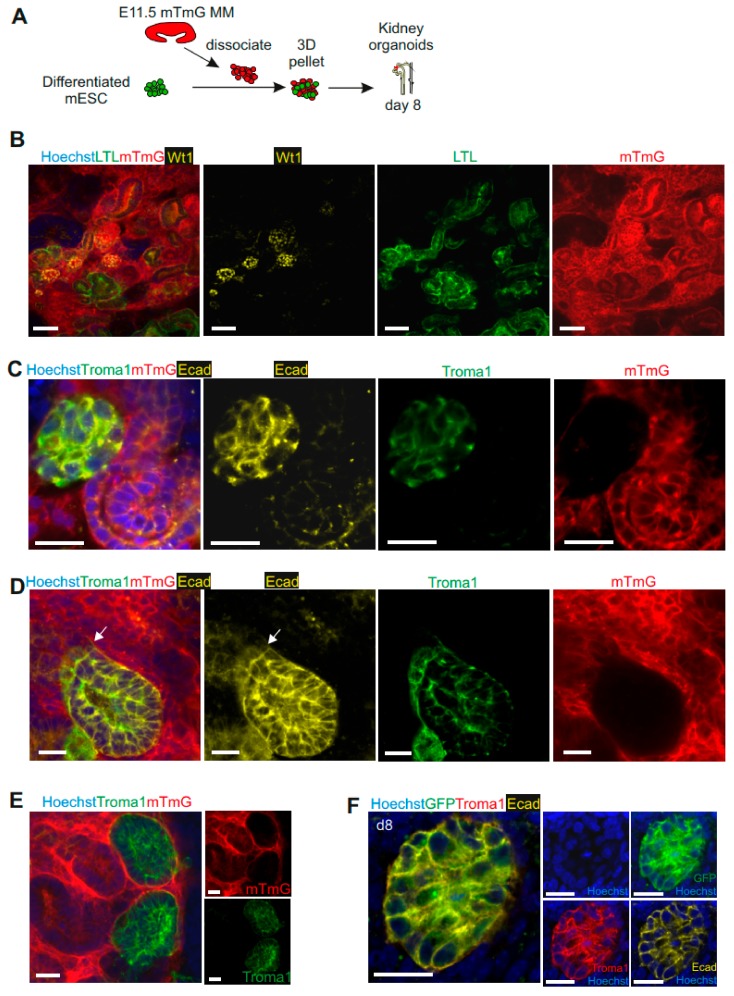
UB structures in kidney organoids are specifically derived from differentiated mESCs. (**A**) Schematic of generation of kidney organoids from mESC-derived UB progenitors with mouse E11.5-dissociated mTmG (td-tomato+) pMM. (**B**) Confocal images of three-dimensional (3D) kidney organoids derived from E11.5 mTmG+ pMM and mESC-derived UB progenitors. Nephron structures such as WT1+ glomeruli and LTL+ proximal tubules were derived from mTmG+ pMM. Scale bars, 50 μm. (**C**) Confocal images of kidney organoids showing ureteric bud structures (Ecad+Troma1+) being generated by mESC-derived UB progenitor cells. Scale bars, 20 μm. (**D**) Confocal images showed Troma1+Ecad+ collecting duct derived from mTmg– cells (mESC-derived UB progenitors) and connected with Troma1–Ecad+ renal tubules (connection marked with an arrow). Scare bars, 20 μm. (**E**) The Troma1+ UB structures derived from mTmG– cells (mESC-derived UB progenitors). Scale bar: 20 μm. (**F**) Troma1+Ecad+ collecting ducts are derived from GFP+ mESCs-derived UB progenitors. Scale bar: 20 μm.

To further confirm that the nephron structures and collecting ducts were derived from pMM- and mESC-derived UB progenitors respectively, we used pMM population isolated from mTmG mice (td-tomato+) to aggregate with differentiated mESCs—unlabeled (Figure 3A). The aggregates formed well-developed nephron structures (WT1+ glomeruli, LTL+ proximal tubules, Ecad+Troma1– distal tubules), which originated from MM cells (mTmG+) (Figure 3B) and Troma1-labeled collecting duct, which was mTmG– and therefore originated from the mESC-derived UB progenitors (Figure 3C–E). We also generated an mESC line with stable expression of the GFP. We differentiated these cells into UB progenitors and used them to induce pMM cells. Immunofluorescence analysis confirmed that the Troma1+Ecad+ collecting ducts were derived from GFP+ mESCs (Figure 3F, Supplementary Figure S4C). Altogether, these data demonstrate that the nephrogenesis occurring in generated kidney organoids is specifically derived from an interaction between the pMM and mESC-derived UB progenitors.

### 3.4. mESC-Derived UB Progenitor Cells Integrated into the 3D Ureteric Bud Structures

**Figure 4:**
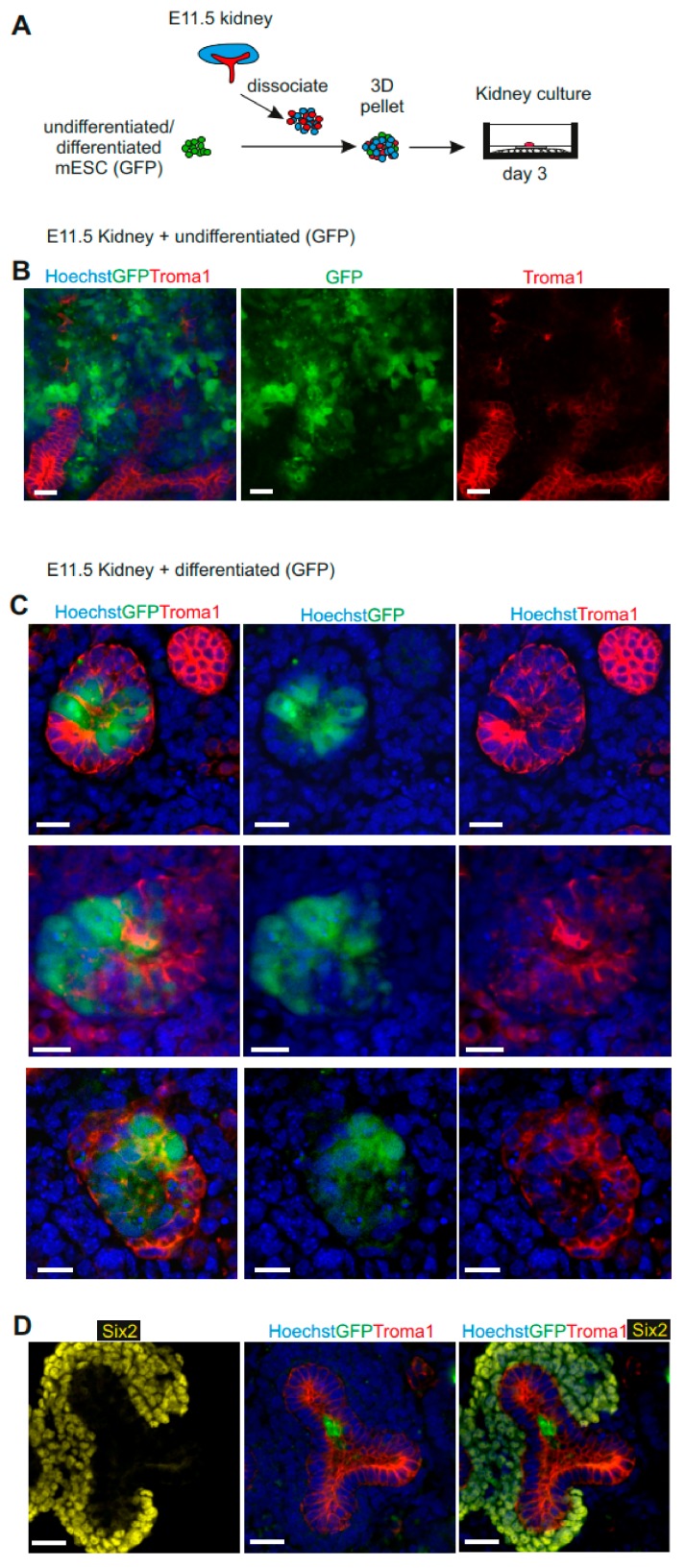
Mouse ESC-derived UB progenitor cells form anembryonic UB in 3D organ culture in vitro. (**A**) Schematic of kidney organoid generation from mESC-derived UB progenitors with mouse E11.5-dissociated kidney rudiments. (**B**) Immunofluorescence analysis demonstrating random localization of undifferentiated mESCs in organ co-cultures. Scale bars, 20 μm. (**C**) Immunofluorescence analysis demonstrating mESC-derived UB progenitors integrated into the UB structures and enhanced chimeric ureteric bud formation. Scale bars, 20 μm. (**D**) Confocal image showing localization of mESC-derived UB progenitor cells (GFP+) in Troma1+ UB structures of the chimeric organoid and not in the Six2+ nephron progenitor’s region. Scale bars: 20 μm.

In order to identify the mESC-derived UB progenitors from wild-type UB cells in chimeric organoids, and to ensure that they will only generate UB cells, we generated an organoid with an mESC-GFP line. We dissociated a whole E11.5 embryonic kidney rudiment (MM and UB) and mixed it with undifferentiated mESC or mESC-derived UB progenitors generating chimeric kidney organoids [19,21,27] (Figure 4A).

The undifferentiated mESCs aggregated with dissociated embryonic kidney rudiments randomly localized within the organoids and had a negative effect on nephrogenesis. The undifferentiated mESC had formed groups within the renal structures; however, there were only a few structures, and very few Troma1+ ureteric buds could be observed (Figure 4B). When we aggregated the mESCs-derived UB progenitors with the dissociated embryonic kidney, we noticed much more robust tubulogenesis and numerous chimeric ureteric bud structures (Figure 4C). The UB progenitor cells did not interfere with renal development, but efficiently and specifically integrated into the mouse ureteric buds, as indicated by Troma1 and GFP+ staining (Figure 4C and D). Moreover, the immunostaining of day three organoids with nephron progenitor’s marker, Six2, presented integration of mESC-derived UB progenitors (GFP+) into the UB structure (Figure 4D). Together, these data demonstrate that mESC-derived UB progenitors do integrate well into ureteric bud structures in chimeric renal organoids and are able not only to form UB de novo, but also generate chimeric structures.

### 3.5. Kidney Organoids as Models to Study Kidney Development and Drug Toxicity

After transfer of the 3D chimeric organoids to organ culture, they self-organized and generated a well-structured kidney organoid with proper nephron and collecting duct structures. As patterning of the nephron into its different segments begins at the renal vesicle stage during development [29,30], we postulated that developmental patterning could be changed by chemical modulation of these endogenous signals. A previous report revealed that Notch signaling is required for proximal tubule fate acquisition in a mammalian nephron [31]. We therefore treated the organoids (pMM cells mixed with mESC-derived UB progenitor cells in a ratio of 3:1, Figure 2A) with a Notch signaling inhibitor, *N*-*S*-phenyl-glycine-*t*-butyl ester (DAPT). DAPT leads to suppression of proximal tubules’ formation in the human nephron organoid culture [4,31,32]. We added DAPT to the culture medium from day 3 to 11 of 3D organoid culture. Immunofluorescence analysis of the cells demonstrated that with the DAPT treatment, formation of glomerulus and ureteric bud ducts was normal, but development of proximal tubules was severely suppressed (Supplementary Figure S5A).

In order to use stem cell-derived kidney organoids for disease modelling and drug screening, they need to present functional maturation of the nephrons within the organoids. To test whether these organoids could be used to study kidney injury and toxicity in vitro, we focused on drug-induced nephrotoxicity, which has been shown as an important cause of acute kidney injury in hospitalized patients [33]. We treated the chimeric kidney organoids with gentamicin, a commonly used antibiotic with well-established proximal tubular toxicity, after nine days of organ culture for 48 h [34]. We also treated organoids with another nephrotoxicant, cisplatin, from day 10 for 24 h. Cisplatin induces caspase-mediated acute apoptosis of proximal tubular cells in the kidney [35]. Whole-mount immunostaining of control organoids with caspase 3 showed random apoptotic interstitial cells; however, both gentamicin and cisplatin induced acute apoptosis in LTL+ proximal tubules (Supplementary Figure S5B). The percentage of apoptotic proximal tubular cells induced by cisplatin increased in a dose-dependent manner: the 5 and 20 µM cisplatin doses mainly affected the proximal tubule compartment, 32% and 62% respectively, while 50 μM cisplatin led to apoptosis of almost all of the proximal tubule cells (≈96%), but was also toxic to other cell types presenting a global type of nephrotoxicity at this concentration (Supplementary Figure S5C).

To summarize, our work shows that mESC-derived UB progenitors induce nephrogenesis in pMM cells, and furthermore, chimeric renal organoids generated from these progenitors show an expected response to toxic chemicals and drugs. We also demonstrated that the kidney organoid system can be used to test nephrotoxicity of drugs and other chemicals in vitro.

## 4. Discussion

Kidney development starts from an interaction between two precursor tissues of the kidney, UB and MM. A major part of the MM cell population comprises the nephron progenitor cells (NPCs) which will differentiate into nephrons, and the ureteric bud will form the collecting duct tree. Protocols for differentiation of mouse and human pluripotent stem cells to renal progenitor cells, and further to self-organized kidney organoids containing nephrons, have been well established [2,3,4,13,36], but current methods of differentiation of pluripotent stem cells, specifically to ureteric bud progenitor cells, need further development [8,19].

Previous studies on generation of ESC-derived UB have shown derivation of a UB-like population by selective induction with metanephric mesenchyme cells [19] or through CHIR99021 treatment for nephron differentiation of both ureteric bud and nephron structures [2]. However, the ESC-derived UB-like cells did not show nephron progenitor induction [19], and therefore, an inter-nephron connection with collecting ducts was lacking [2]. Another group recently published a protocol for derivation of UB structures from PSCs, and generation of kidney organoid composed of mESCs-derived UB aggregated with pMM, or mESCs-derived UB combined with mESC-derived NPC and primary stromal progenitor cells (SPs). Although successful, their protocol requires a knock-in of markers in the PSCs, which later involves sorting of cells for the specific marker [8]. More recently, Mae and colleagues reported a protocol for generation of branching ureteric bud tissues from human pluripotent stem cells (hPSC) with a series of growth factors, but there was no evidence of nephrogenesis [37] (Supplementary Table S2). While these published protocols produce functional kidney organoids, they are technically complex. Here, we reported an establishment of a simple (directed with growth factors), efficient (>90% of Pax2+, Ecad+ and >70% Gata3+ cells), and reproducible differentiation protocol of mESC to ureteric bud progenitor cells. These mESC-derived UB progenitor cells induced pMM cells to undergo nephrogenesis leading to development of well-structured nephrons. These nephrons consisted of glomeruli, proximal tubules, loops of Henle, and distal tubules, and were connected with collecting duct structures generated by mESC-derived UB cells. Moreover, these mESC-derived UB progenitor cells formed a UB de novo when combined with pMM cells, and they generated chimeric structures when combined with kidney rudiment cells (pUB and pMM) (Supplementary Table S2).

While our culturing conditions produced well-functioning kidney organoids, further studies are needed to fine-tune the culturing conditions. Previous studies showed that ROCK kinases are VEGF downstream effectors, which negatively regulate the process of angiogenesis [28]. We added ROCK inhibitor (Y27) to culture expecting an increase in angiogenesis. However, we failed to see a difference between control and Y27-treated samples. Better results could be obtained by supplementing the medium with VEGF, which was already published by Freedman and co-workers, although they still did not observe endothelial cells entering the glomerular tuft in their system [9]. Similarly, development of a vascular glomeruli in organotypic kidney cultures and renal organoids was reported earlier by our research team [38]. It seems that in the absence of the blood flow, the endothelial cells are not able to properly interact with developing nephrons [39] and formation of glomerular vasculature does not proceed further than migration of endothelial cells into the vascular cleft region of an S-shaped stage nephron. Sufficient vascularization of the organoids may be achieved by treatment with angiogenic factors, co-culture with blood vessel organoids [40], by providing flow to the system [15], or with a combination of the aforementioned treatments.

Even though organoids generated by mESC-derived UB progenitor cells did not have a proper vascular network, the developed nephrons did respond to toxicological tests as expected. We and others [4] have shown that the use of gentamicin and cisplatin induces apoptosis in proximal tubular cells. Thus, these organoids present a functional platform to test drug-induced nephrotoxicity.

In summary, we have developed an easy and reproducible protocol for generation of UB progenitors from mESCs. This work generates a strong foundation for in vitro kidney studies, including disease modelling and drug discovery approaches, which are difficult to perform, and require animal models and/or primary cells which may not faithfully recapitulate all features of developmental or disease processes. Given the rapid progress in the field, we hope that in the near future, researchers will be able to generate fully functional nephrons in kidney organoids where the UB and MM parts are derived from PSC. Using these cells will enable generation of not only well-structured nephrons, but also the collecting duct tree. This is the first step for generating high-throughput gene discovery models and advancing tissue engineering for producing organs for transplanting. However, these organoids need to be successfully vascularized and grown to appropriate size. The studies presented here produce new insights into renal pathophysiology and open new avenues for developing new treatment options.

## Supplementary Materials

The following are available online at https://www.mdpi.com/2073-4409/9/2/329/s1. Table S1: Primers (5′-3′) used in the study. Table S2: Summary of available protocols of differentiation of mouse and human PSC to UB linages. Figure S1: Differentiations of mESC into ureteric bud progenitor cells. Figure S2: Differentiated mESC induce embryonic MM to nephrogenesis. Figure S3: Vascularization of the kidney organoids. Figure S4: Characterization of kidney organoids. Figure S5: Kidney organoids model kidney development and injury.
