# Supplementary material for: Mouse Embryonic Stem Cell-Derived Ureteric Bud Progenitors Induce Nephrogenesis
Source: Cells. 2020 Jan 31;9(2):329. doi: 10.3390/cells9020329 (PMC7072223; doi:10.3390/cells9020329)

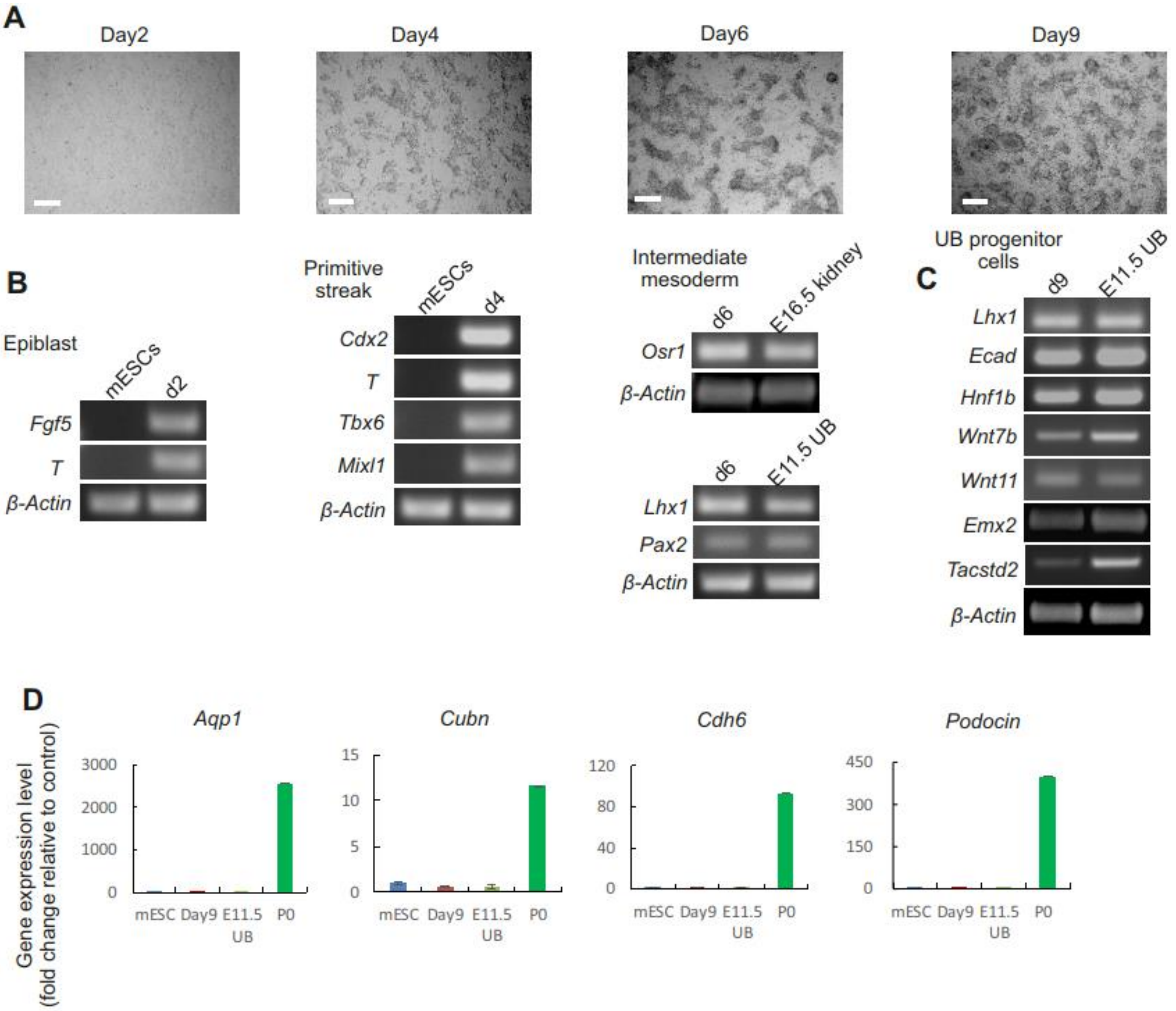

Figure S1: Differentiation of mESCs into ureteric bud progenitor cells. (A) Brightfield images of mESCs in monolayer (2D) cultures during differentiation into UB progenitor cells. Consecutive days are shown. Scale bars, 200 µm. (B) RT-PCR displays mESC differentiation into epiblasts but not to extraembryonic endoderm at day 2, primitive streak but no ectoderm at day 4 and intermediate mesoderm but no ectoderm). β-actin was used as a housekeeping gene, E16.5 kidney, E11.5 UB and mESC were used as a control. Cell-specific markers: *Afp*, extraembryonic endoderm; *Pax6*, ectoderm; coexpression of *Pax6 and Sox1*, ectoderm. (C) RT–PCR at day 9 of differentiation showing the expression of markers of UB progenitors while ectodermal marker *Pax6* was not detected; E11.5 UB was used as positive control. (D) qPCR graphs show lack of nephron specific marker expression in day 9 differentiated cells.

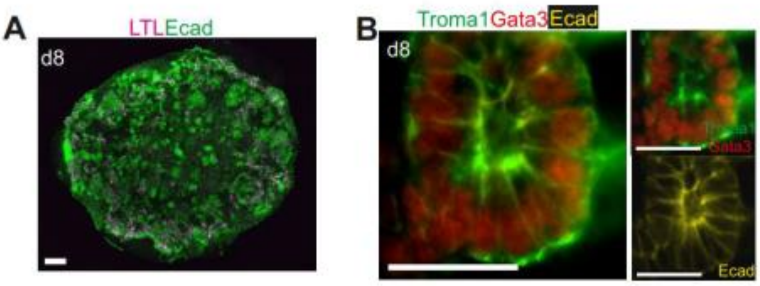

Figure S2: Differentiated mESCs induce embryonic MM to nephrogenesis. (A) Tile scan immunofluorescence of a whole kidney organoid displaying complexity of tubular structures. The proximal tubules marked with LTL (magenta), nephron tubules and collecting ducts labelled with Ecad (green). Scale bar, 200 µm. (B) Confocal images of collecting duct structure. Scale bare 20 µm.

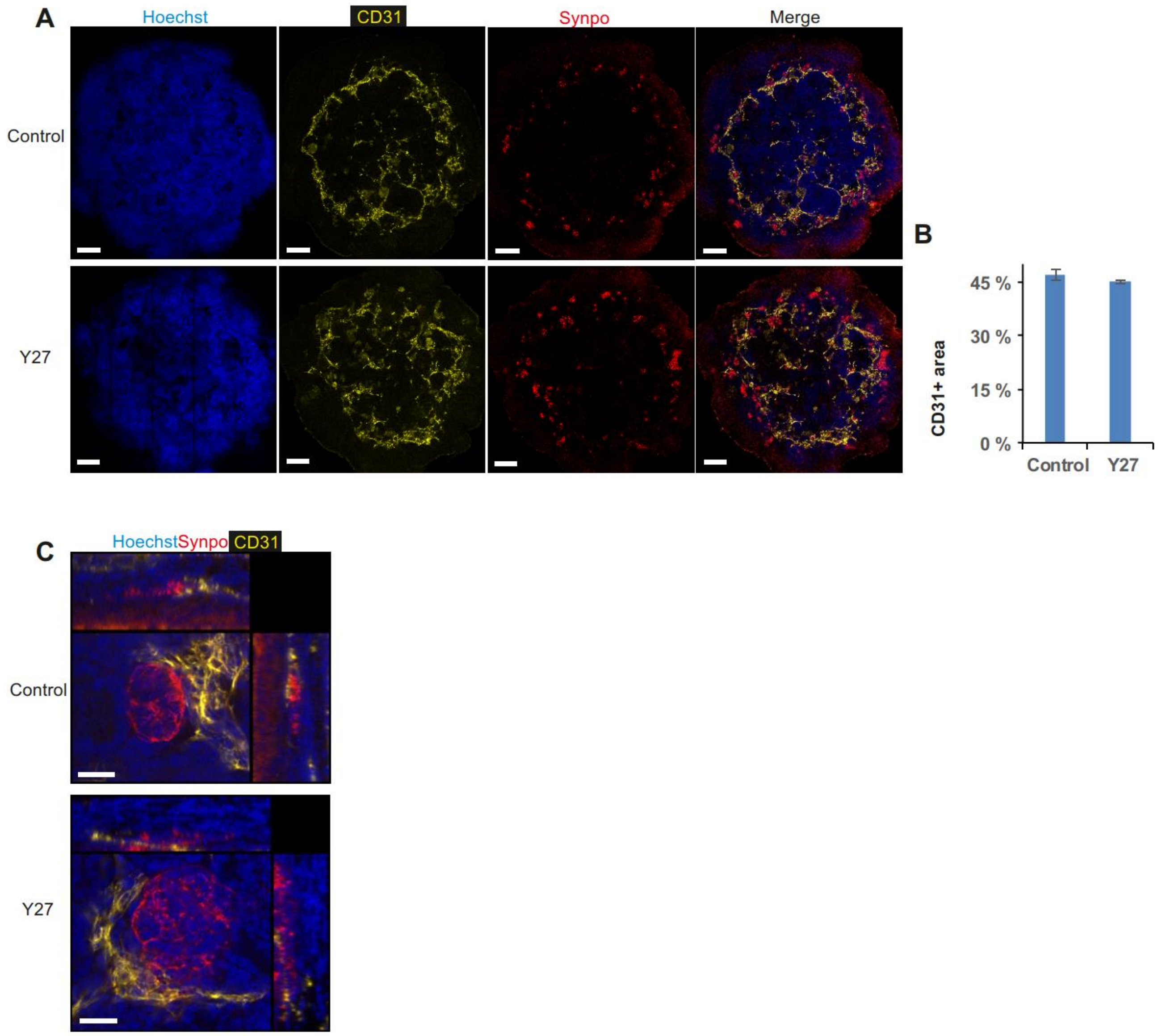

Figure S3: Vascularization of the kidney organoids. (A) Tile scan immunofluorescence of the whole kidney organoids displaying vascularization network treated with Rock inhibitor (Y27) or without (Control). Stains: Hoechst, DNA; glomeruli, Synpo, endothelial cells, CD31Scale bars, 200 µm. (B) Percentage of the total culture area occupied by cells expressing CD31, n=6 experiments (+/- S.E.M.). (C) Confocal multiple image projection (MIP) showing glomeruli with endothelial cells in organoids treated with Y27 or without treatment (control). Scale bars, 20 µm.

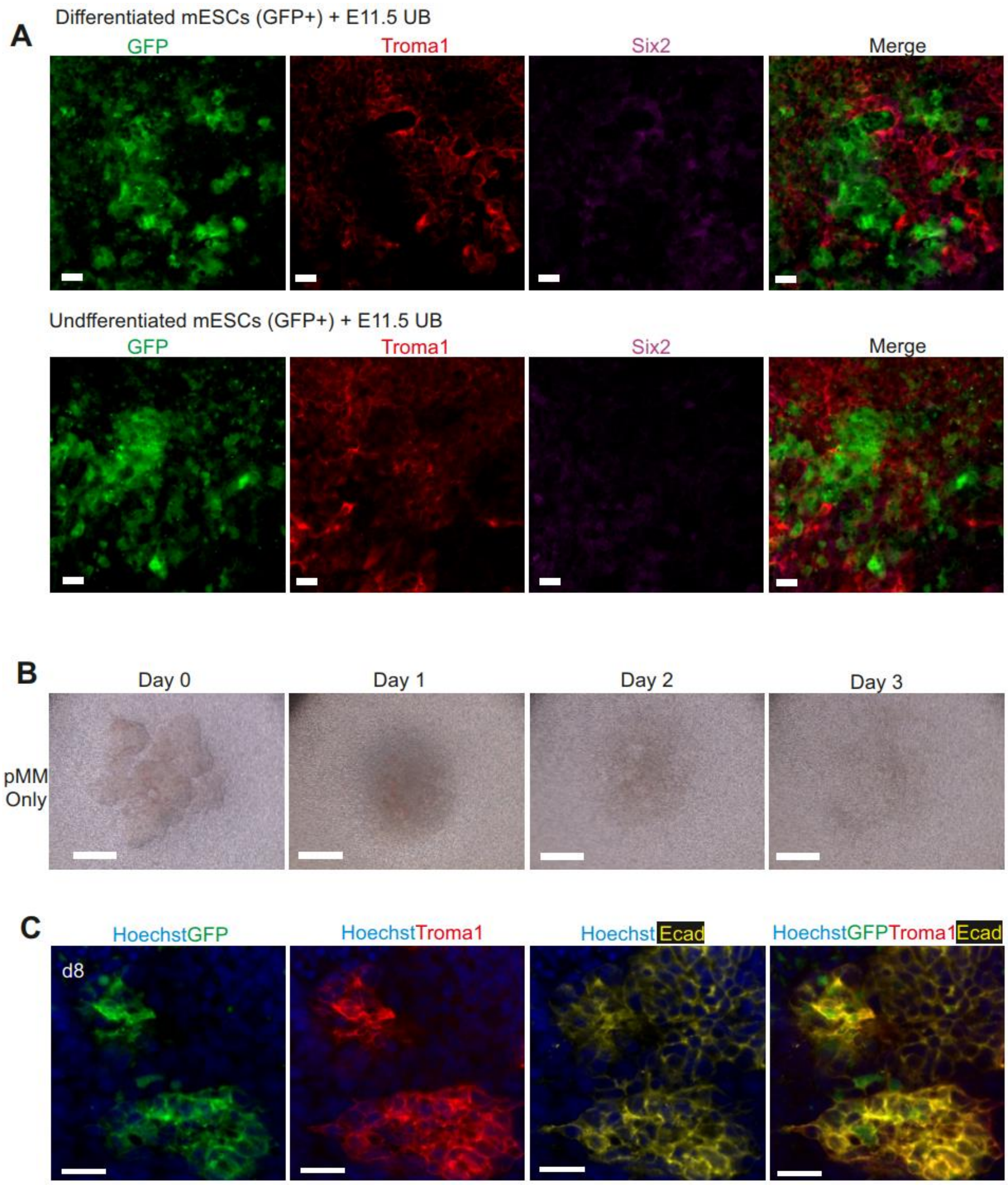

Figure S4: Characterization of kidney organoids. (A) Confocal images showed no visible signs of *in vitro* nephrogenesis at day 3 of culture when dissected mouse embryonic ureteric bud (UB) cells were co-cultured with differentiated or undifferentiated GFP+ mESCs. Lack of nephron progenitors leads to lack of nephrogenesis. Collecting duct labelled with Torma1 (red), mESC-derived UB cells (GFP, upper panel) and undifferentiated mESCs (GFP, lower panel), nephron progenitors marked with Six2 (purple). Scale bars, 20 μm. (B) Primary MM (pMM) cultured alone on a 3D Trowell culture died after 3 days (n=3). Scale bars, 500 μm. (C) Immunostaining of Troma1+Ecad+ chimeric collecting ducts generated by GFP+ mESCs-derived UB progenitors and pUB in chimeric kidney organoids. Scale bars, 20 μm.

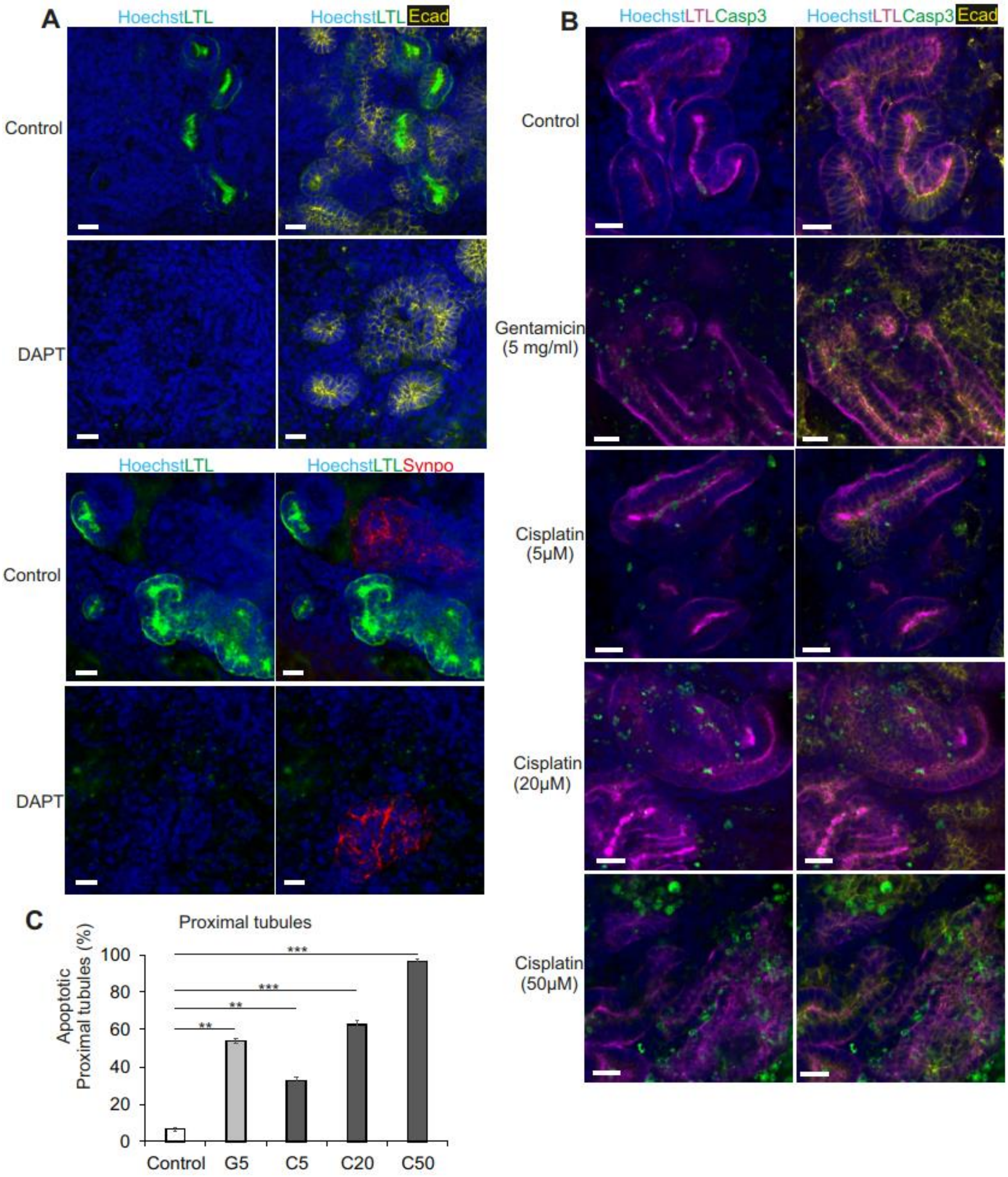

Figure S5: Kidney organoids model kidney development and injury. (A) Representative images of immunohistochemistry of a kidney organoid showing lack of proximal tubule structures when treated with 10 µM DAPT from day 3 to 11. Notch inhibition suppressed the proximal tubule (LTL+, green) formation, but the UB (Ecad+, yellow) and glomerular (Synpo+, red) structures developed normally. n = 5; Scale bars, 20 µm. (B) Representative immunohistochemistry images of renal structures treated with gentamicin (5 mg/ml) from day 9 to 11 (48 hours) or cisplatin (5, 20, 50 µM) from day 10 to 11 (24 hours). Apoptotic cells were detected by cleaved caspase 3 antibody-staining (Casp3). n = 6; Scale bars, 20 µm. (C) Quantification of number of apoptotic proximal tubular cells in kidney organoids treated with gentamicin (G) (5 mg/ml) and cisplatin (C) (5, 20, 50 µM) at indicated doses. Data are expressed as mean ± s.e.m. (n=5); ** - $p<0.01$; *** - $p<0.001$.

Table S1: Primers (5′-3′) used in the study.

Gene name	Forward primer sequence	Reverse primer sequence
β-Actin	GATCTGGCACCACACCTTCT	GGGGTGTTGAAGGTCTCAAA
GADPH	CATCAGTCCTTCCACGATACCA	CCTGCACCACCAACTGCTTA
Fgf5	GCTGTGTCTCAGGGGATTGT	CACTCTCGGCCTGTCTTTTC
T	GCTTCAAGGAGCTAACTAACGAG	CCAGCAAGAAAGAGTACATGGC
Mixl1	ACGCAGTGCTTTCCAAACC	CCCGCAAGTGGATGTCTGG
Tbx6	ATGTACCATCCACGAGAGTTGT	GGTAGCGGTAACCCTCTGTC
Cdx2	AGCTGCTGTAGGCGGAATGTATG	TCAGTGACTCGAACAGCAGCAA
Osr1	GAGCGACCTTACACCTGTGA	GTCTTGTGGACAGCGAGAGT
Lhx1	CAGTGTCGCCAAAGAGAACA	TGAGACGTTGGCACTTTCAG
Hoxb7	GACTTGGCGGCCGAGAGTAA	CCGAGTCAGGTAGCGATTGTAGTG
Emx2	GATATCTGGGTCATCGCTTCCAA	GCTCCCACCACGTAATGGTTC
Wnt11	TGTGCGGACAACCTCAGCTAC	ATGGCATTTACACTTCGTTTCCAG
Sox9	AGGAAGCTGGCAGACCAGTA	TCCACGAAGGGTCTCTTCTC
Ecad	CACCGATGGTGAGGGTACACAG	GGCTTCAGGAATACATGGACAAAGA
Hnf1b	CGCGGTGACTCAGCTACAGAAC	TCACCAGGCTTGCAGTGGAC
Wnt7b	TACCTAAGTTCCGCGAGGTG	AGGCTTCTGGTAGCTGCGTA
Tacstd2	ACTGTACATGCCCCACCAAC	GCAGGCACTTGGAAGTTAGC
Pax2	CGCCGTTTCTGTGACACACAATC	TGCTTGGGACCAAACACAAGGTG
Ret	TTCTGAAGACAGGCCACAGGA	CACTGGCCTCTTGTCTGGCT
Wnt9b	TGGCTTTCGTGAGCATGGAG	AAAGACAGCCACGGTGTGGTAA
Calb1	CCTTTGTGGATCAATATGGACAGA	TCAGTTGCTGGCATCGAAAG
Aqp1	AGGCTTCAATTACCCACTGGA	GTGAGCACCGCTGATGTGA
Cubn	CACTTTAGGTTGTGGTGGAACA	TTGCTGTCAAAGCTAATCTCCC
Cdh6	CAGCCCTACCCAACTTTCTCA	GAACGGCTCAGCTCATTCC
Podocin	GACCAGAGGAAGGCATCAAGC	GCACAACCTTTATGCAGAACCAG

Table S2: Summary of available protocols of differentiation of mouse and human PSC to UB lineages.

		Our protocol	Taguchi et al., 2017	Taguchi et al., 2017	Takasato et al., 2015	Xia et al., 2013	Mae et al., 2018
Cell type		mESC	mESC	hiPSC	hESC/ hiPSC	hESC/ hiPSC	hESC/ hiPSC
Differen- tiation condition	Basic differentiation medium	Advance RPMI 1640	IMDM/F12	IMDM/F12	APEL	DMEM/ F12	Essential 6
	Specific factors (PSC differentiated to IM)	Activin A	Activin A	Activin A	CHIR99021	Activin A	Activin A
		FGF2	BMP4	BMP4	FGF9	FGF2	BMP4
		CHIR99021	CHIR99021	CHIR99021		Retinoic Acid	CHIR9902 1
		Noggin	Retinoic Acid	Retinoic Acid		BMP4	LDN1931 89
			FGF9	FGF9		BMP2	A83-01
			SB431542	LDN193189			FGF8
				SB431542			TTNPB
	Specific factors (IM differentiated to UB)	FGF9	Retinoic Acid	Retinoic Acid	CHIR99021		CHIR9902 1
			CHIR99021	CHIR99021	FGF9		LDN1931 89
			FGF9	FGF9			FGF8
			Y27632	Y27632			GDNF
			GDNF	GDNF			FGF1
				LDN193189			TTNPB
				FGF1			Thiazoviv in
FACS sorting step		no	yes, FACS sorting of the Cxcr4+/Kit+ cells	yes, FACS sorting of the CXCR4+/KI T+ cells	no	no	no
Generation of UB de novo		yes	yes	yes	yes	no	yes
Collecting duct connect with nephron tubule		yes	yes	not shown	yes	not shown	not shown
Source of MM (aggregate to form organoid)		E11.5 MM	mESC- derived NPC+E11.5 stromal cells or E11.5 MM	no	hPSC- derived renal progenitors	no	no
Integration of UB cells		yes, Integrated into E11.5 mouse UB on organ culture	Yes, Integrated into E11.5 mouse UB on organ culture	not shown	not shown	yes, Integrate d into E11.5 mouse UB on organ culture	not shown

Full Gel images

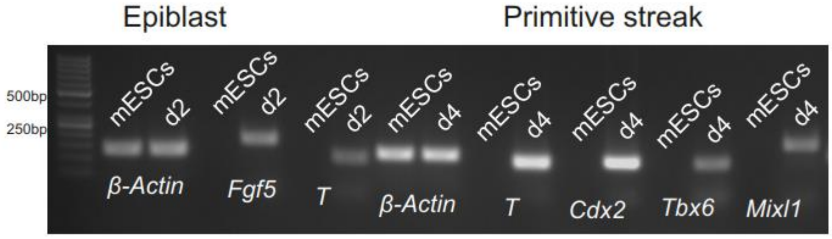

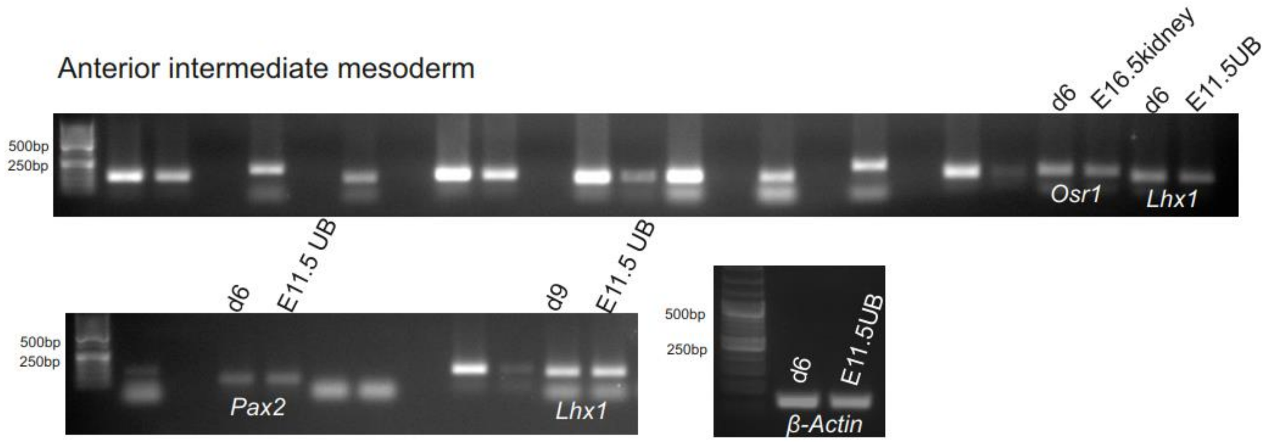

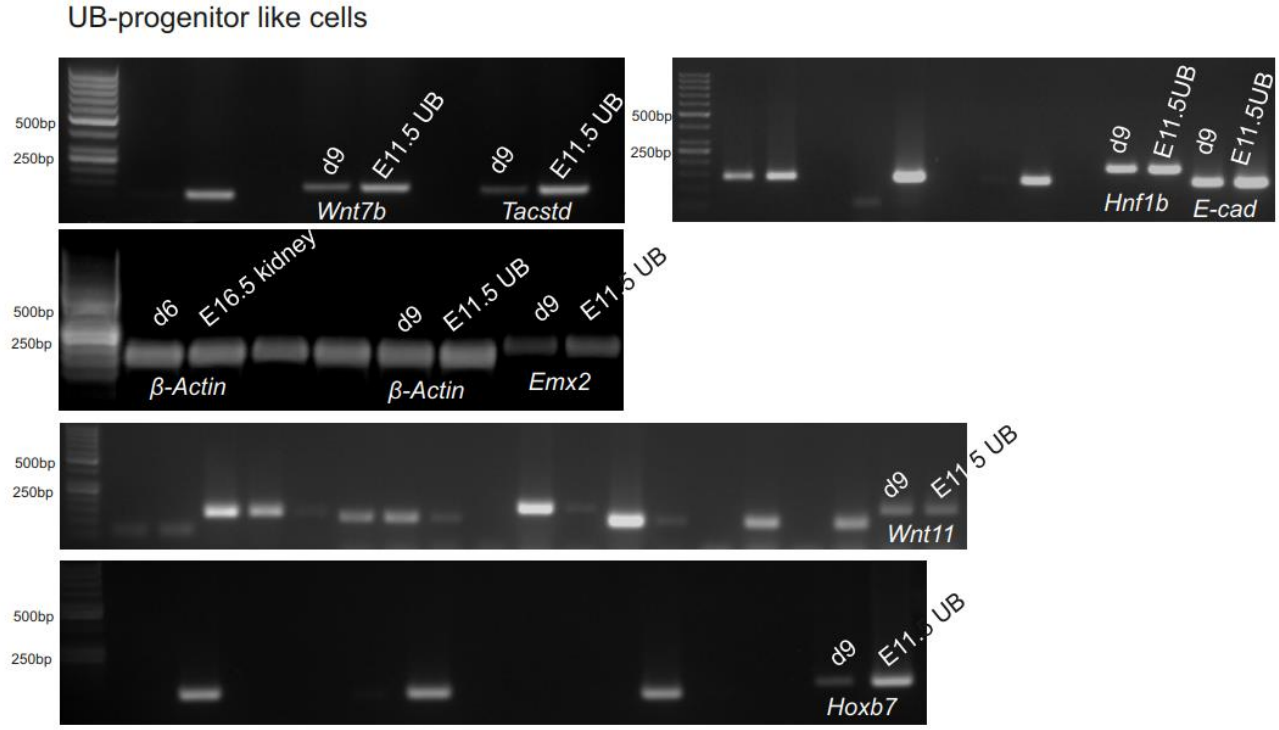

Supplement: Supplementary file 1 [file cells-09-00329-s001.pdf]
